# The human microbiome reshapes the breast cancer immune-metabolic-hormonal microenvironment

**DOI:** 10.3389/fimmu.2026.1774211

**Published:** 2026-04-15

**Authors:** Qianheng Wang, Furong Zhang, Yue Xin, Rui Yang, Zimei Tang, Zhenghao Wu, Jie Ming

**Affiliations:** Department of Breast and Thyroid Surgery, Union Hospital, Tongji Medical College, Huazhong University of Science and Technology, Wuhan, China

**Keywords:** breast cancer, gut-breast axis, immune regulation, microbiome, precision medicine, tumor microenvironment

## Abstract

Beyond established risk factors such as genetics and hormones, the human microbiome has emerged as a pivotal player in breast cancer pathogenesis. This review delineates the technological evolution in breast microbiome research, spanning traditional culture methods to high-throughput sequencing and cutting-edge spatial omics. We elucidate the role of the gut-breast axis in modulating breast cancer development through its influence on estrogen metabolism, immune responses, and microbial metabolites. Furthermore, we analyze the distinctive compositional features of the intratumoral microbiota and their dual, context-dependent roles in promoting invasion, inducing immunosuppression, and driving metabolic reprogramming within the tumor microenvironment. Novel microbiome-based therapeutic strategies, including targeted microbiota depletion, engineered microbial therapeutics, and dietary interventions, are summarized. Finally, we discuss the translational potential of microbiome research in refining breast cancer risk prediction, evaluating treatment responses, and advancing personalized prevention and treatment strategies, ultimately contributing to improved patient outcomes.

## 1 Introduction: from sterile tissue to “microbe-host interactions”

Breast cancer (BC) persists as the principal cause of cancer-related mortality among women worldwide, maintaining elevated incidence rates alongside substantial molecular and clinical heterogeneity (1). This multifaceted complexity presents considerable challenges for accurate diagnosis, effective therapeutic intervention, and precise prognostic assessment (2). While conventional etiological frameworks emphasize genetic predisposition, hormonal exposure, and lifestyle factors as primary determinants, these elements collectively fail to comprehensively elucidate the pathogenesis of all breast cancer subtypes (3). The recent paradigm shift, catalyzed by advancements in high-throughput sequencing and multi-omics analytical platforms, has progressively unveiled the instrumental role of the human microbiome in oncogenesis across multiple malignancies (4). Currently, the dynamic interplay between microbial communities and host systems—encompassing immunomodulation, metabolic reprogramming, endocrine signaling, and therapeutic response mechanisms—represents an emerging frontier in breast cancer research, creating unprecedented opportunities for innovative approaches to disease prevention, diagnostic refinement, and treatment optimization.

The long-held dogma of the “sterile breast” was fundamentally challenged in 2016 by two seminal studies. Chan et al. provided initial evidence by detecting bacterial DNA via 16S rRNA amplicon sequencing in nipple aspirate fluid from 23 breast cancer survivors, reporting a significant enrichment of the genus *Alistipes (*5). Concurrently, Hieken and colleagues conducted the first comprehensive 16S rRNA gene sequencing analysis on 78 aseptically collected breast tissue specimens. Their work not only confirmed the existence of a distinct microbial community resident in breast tissue but also delineated its unique compositional profile, which significantly differed from the microbiota of breast skin, skin swabs, and oral swabs. These pivotal findings collectively laid the foundational framework for the field of breast cancer microbiome research. Furthermore, they revealed specific microbial alterations associated with breast carcinogenesis, notably a significant upregulation of low-abundance taxa such as *Fusobacterium* and *Lactobacillus* in malignant tissues compared to their benign counterparts (6). By definitively overturning the sterile breast paradigm, these landmark investigations inaugurated a novel and fertile area of inquiry into the role of the microbiome in breast cancer.

Subsequent advancements in gut microbiome research precipitated a paradigm shift, revealing that the microbiological landscape of breast cancer is not localized but is intricately interconnected with systemic microbial communities, most notably the gut microbiota, via complex signaling networks. This recognition gave rise to the “Gut-Breast Axis” concept, which mechanistically links gut microbial dynamics to estrogen metabolism, host immune responses, and other cancer-relevant processes in breast cancer patients. Concurrently, spatial metagenomic profiling of the tumor microenvironment has unveiled a remarkable prevalence of intracellular microbiota, with over 80% of microbes residing within cancer and immune cells. Critically, the composition of these intratumoral communities exhibits direct correlations with tumor subtypes and responses to immunotherapy, providing spatially resolved evidence for functional “microbe-host” co-localization (7). The convergence of these insights has crystallized a multidimensional network of “local microbiota-systemic microbiota-host immunity-metabolism-hormones,” thereby establishing “microbiota-host interactions” as a transformative research paradigm in breast oncology.

**Figure 1:**
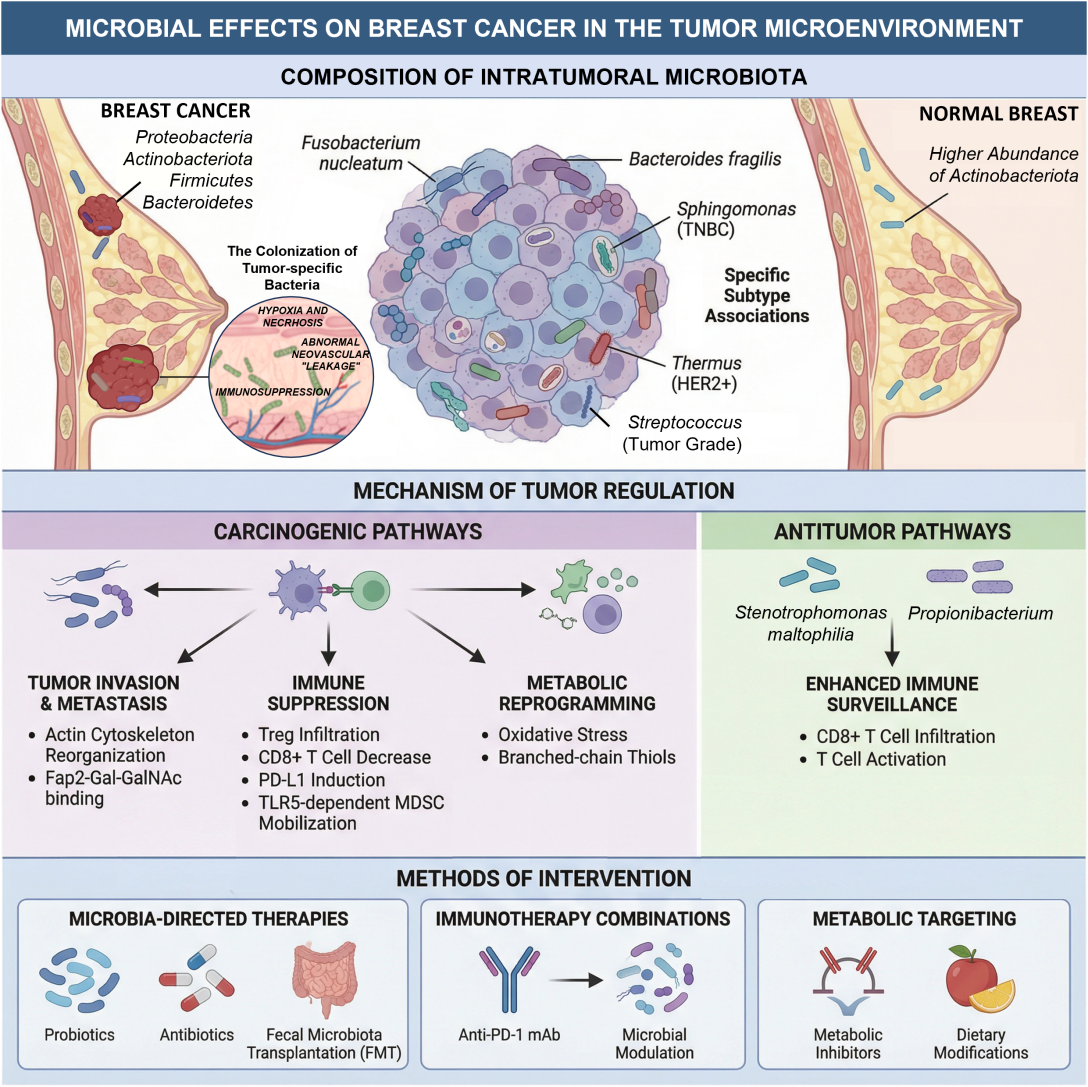
Breast cancer and normal breast tissue differ in intratumoral microbiota composition, and the composition infects the mechanism of tumor regulation. The methods of intervention have also been proposed and applied.

This evolving paradigm not only deepens our mechanistic understanding of breast oncogenesis but also illuminates novel avenues for precision oncology. Accordingly, this review will critically synthesize current knowledge by exploring three pivotal themes: technological advancements in microbial profiling, the mechanistic underpinnings of the gut-breast axis, and the functional roles of microbes within the breast tumor microenvironment. By delineating the pathophysiological mechanisms and their translational implications, we aim to establish a conceptual framework that informs future research and refines precision medicine strategies for breast cancer (**Figure 1**).

## 2 Innovations in microbial detection technologies and clinical translation

### 2.1 Traditional culture → 16S rRNA sequencing → metagenomic analysis → spatial omics

The trajectory of breast cancer microbiome research over the past twenty years is defined by a transition from inferential population-level profiling to the precise, *in situ* characterization of microbial communities at single-cell resolution—a leap driven by progressive technological innovation.

The foundational investigations relied upon selective culture media and biochemical identification. While these methods provided the first crucial evidence of viable bacterial communities within breast biopsy tissues, they were capable of recovering less than 1% of the actual breast microbiota (6). Inherent culture biases and stringent anaerobic requirements resulted in a severe underestimation of low-abundance and fastidious bacteria, thereby limiting insights into microbiota-tumor phenotype associations.

The advent of 16S rRNA high-throughput sequencing in the early 21st century effectively circumvented the cultivation bottleneck, unveiling significant differences in microbial composition across distinct breast cancer subtypes. Employing V3–V4 region sequencing, Chan et al. were among the first to identify differentially abundant genera, including *Alistipes* and *Sphingomonas*, in the nipple aspirate fluid of breast cancer patients (5). Subsequently, Costantini et al. demonstrated that 16S rRNA profiles from core needle biopsy (CNB) specimens showed no significant divergence from those of surgical resection samples, thereby establishing a methodological foundation for minimally invasive sampling (8). One study, based on a large-scale comparative analysis of sequencing nine hypervariable regions (V1V2, V2V3, V3V4, V4V5, V5V7, and V7V9) of the 16S rRNA gene, suggested that V1V2 amplicon sequencing is better suited for profiling the normal breast microbiome (9). A principal limitation of this approach, however, is its provision of taxonomic classification generally only to the genus level, its inability to resolve strain-specific functional capacity, and its susceptibility to artifacts from primer bias and 16S rRNA gene copy number variation.

Shotgun metagenomic sequencing represents a substantial advance by enabling strain-level resolution and comprehensive functional gene annotation through the untargeted sequencing of all microbial DNA within a sample. This methodology revealed, for instance, elevated abundances of *Clostridiales* and *Faecalibacterium* in the gut microbiota of postmenopausal breast cancer patients. Crucially, it identified a concomitant enrichment of β-glucuronidase genes, establishing an initial functional link between specific gut microbial communities and the reactivation of estrogens (10). Furthermore, metagenomics has facilitated the identification of potential pathogenicity islands, antibiotic resistance genes, and virulence factors in breast tissue, providing a genetic blueprint for mechanistic inquiry. This technique remains constrained by its reliance on homogenized samples, which erases all spatial and cell-specific information. Additionally, significant methodological caveats have been identified, including the misclassification of millions of host-derived DNA sequence reads as microbial due to errors in processing pipelines and databases, as well as the generation of artifactual microbial features during batch-effect correction procedures, even when such clusters are absent from the raw data (11).

Emerging spatial omics technologies—encompassing spatial transcriptomics, *in situ* RNA-Scope, and high-resolution mass spectrometry imaging—directly address this limitation by preserving the anatomical context of the tissue. These platforms allow for the precise co-localization of microbial abundance with host transcriptomic, proteomic, and metabolomic profiles, thereby revealing a complex, three-dimensional “microbe-host-microenvironment” interaction network. Research utilizing these tools has demonstrated that over 80% of the bacterial load within breast cancer tissues resides intracellularly, specifically to breast cancer subgroup or the entire pan-cancer, primarily within cancer cell cytoplasms and CD68^+^ macrophages (7). While spatial omics technologies are still maturing, they offer a transformative perspective for understanding the nuanced roles of microbes within the breast cancer microenvironment.

Concurrently, the development of live-cell tracing and multimodal imaging, such as the application of acoustic reporter genes (ARGs) combined with ultrasound-optical-magnetic imaging, permits the real-time, *in vivo* tracking of bacterial colonization and functional gene expression. Novel bacterial ARG constructs have maintained a 9-fold signal-to-noise ratio at tissue depths of up to 3 cm, successfully enabling the monitoring of the 72-hour dynamic distribution of oncolytic E. coli within a 4T1 mouse model of breast cancer (12). This technology is currently being integrated with clinical imaging modalities like PET-MRI to develop non-invasive pharmacodynamic endpoints for therapeutic translation. Although the clinical application of live bacterial tracing faces considerable challenges, it provides a powerful suite of tools for interrogating the dynamic behavior of microorganisms throughout breast cancer progression (**Table 1**).

**Table 1:** Comparison of methodological approaches in breast cancer microbiome research.

Technical approach	Fundamental principles	Advantages	Limitations	Representative studies
Traditional Culture	Isolation and culture of microorganisms using selective media and biochemical identification methods	First demonstrated the presence of viable bacteria in breast tissue; laid the foundation for subsequent	Recovered <1%6 of bacteria; severely underestimated low-abundance/difficult-to-culture bacteria; introduced culture bias; struggled to explain microbial-tumor phenotype associations	Hieken et al, 2016 (6)
16SrRNA amplicon sequencing	High-throughput sequencing of specific variable regions of bacterial 16SrRNA genes	Overcomes cultivation limitations; reveals microbial community structure; demonstrates microbial composition differences across breast cancer subtypes: supports minimally invasive sampling	Low resolution (typically genus-level only); cannot resolve strain functionality; susceptible to primer bias and copy number variation	Chan et al, 2016 (5)
				Costantini et al, 2018 (8)
Metagenomic sequencing	Shotgun sequencing of all microbial DNA in a sample	Strain-level resolution; functional gene annotation (e.g., KEGG pathways); identification of potential pathogenicity islands, antibiotic resistance genes, virulence factors: linking microbial functions to phenotypes (e.g., estrogen reactivation)	High cost: complex data analysis; remains a “homogenized” sample, losing spatial and cell-specific information	Zhu et al, 2018 (10)
Spatial Omics	Spatial localization of microbial and host molecules in situ	Preserves spatial information: enables co-localization of microbial and host transcriptomes/proteomes/metabolomes; reveals three-dimensional “microbe-host microenvironment” interaction networks provides coordinates for functional validation	Technology still evolving; extremely high cost; complex data analysis	Nejman et al, 2020 (7)
Live-cell tracing and multimodal imaging	Real-time tracking of live Bacteria dynamics *in vivo* using reporter genes and multimodal imaging	Real-time, dynamic, *in vivo* monitoring; capable of tracking bacterial colonization, distribution, and functional expression; holds clinical translation potential (e.g., non-invasive pharmacodynamic assessment)	Currently primarily used in preclinical research; high technical complexity; requires further validation for clinical translation	Hurt et al, 2023 (12)

### 2.2 Clinical translation: standardized sampling, quality control, and ai diagnostic models

Microbiome research has made significant strides in breast cancer, yet clinical translation faces numerous challenges. Standardized sampling, quality control, and the development of artificial intelligence (AI) diagnostic models are critical for achieving this translation.

Microbiome composition and function are highly susceptible to sampling methods, sample handling, storage conditions, and analytical workflows. To ensure reliable and reproducible results, strict standardized sampling protocols must be established. For breast tissue samples, sterile collection is essential, with clear differentiation between tumor tissue, adjacent normal tissue, and distal normal tissue to prevent cross-contamination and interference from background microorganisms (8, 13, 14). For gut microbiome samples, strict standardized procedures are also required for fecal sample collection, storage, and transport. Furthermore, rigorous quality control is essential throughout high-quality DNA/RNA extraction, library preparation, sequencing depth, and bioinformatics analysis steps-–including denoising, chimera removal, and taxonomic assignment-–to minimize technical errors and batch effects (15). Also, variations in bioinformatics pipelines—including clustering thresholds—are critical sources of technical heterogeneity that must be considered when comparing findings across different studies. Previous researchers have emphasized that variations in microbiome characteristics across studies may be linked to environmental and population location, further highlighting the importance of standardization and quality control (14).

The development of artificial intelligence (AI) diagnostic models provides powerful tools for the clinical application of microbiome data. Due to the high dimensionality, complexity, and nonlinearity of microbiome data, traditional statistical methods struggle to fully unlock its potential value. Machine learning and big data analytics can identify specific microbial biomarkers or patterns associated with breast cancer occurrence, progression, prognosis, and treatment response from vast microbiome datasets. Research indicates that a novel breast cancer subtype system, established using machine learning methods based on gut microbiome-related metabolic pathways, host gene expression profiles, and patient prognosis, can identify “challenging breast cancers” with more gene mutations and a more complex immune microenvironment. Its scoring index shows a significant negative correlation with patient prognosis and effectively predicts response to neoadjuvant therapy (16). A model integrating intratumoral microbiome and clinical-pathological features demonstrated robust predictive power for predicting pathological complete response (pCR) to neoadjuvant chemotherapy-immunotherapy (Chemo-IM) in triple-negative breast cancer patients (17). These studies demonstrate that AI models can integrate multi-omics data to identify complex microbiome patterns, thereby providing personalized guidance for breast cancer early diagnosis, risk assessment, prognosis evaluation, and treatment selection.

However, AI model development requires large-scale, high-quality clinical data for training and validation, alongside addressing interpretability, generalization capabilities, and ethical concerns to achieve true clinical translation. Future research should focus on establishing multicenter, prospective cohorts to validate these models’ robustness and clinical utility.

## 3 Advances in gut-breast axis research

### 3.1 Concept and clinical evidence of the gut-breast axis

The Gut-Breast Axis refers to a complex network through which the gut microbiota and its metabolites influence breast tissue physiology, breast cancer development, progression, and treatment response via multiple pathways. This concept is grounded in growing evidence linking gut dysbiosis to increased breast cancer risk, accelerated disease progression, and impaired treatment efficacy.

Multiple studies have revealed differences in gut microbiota composition between breast cancer patients and healthy individuals, characterized by reduced diversity or enrichment/depletion of specific microbial populations. Metagenomic analyses indicate decreased gut microbial diversity in postmenopausal breast cancer patients, alongside enrichment of certain microbial groups (10). *Bifidobacteria* and *Lactobacilli* levels in the intestines of breast cancer patients are significantly lower than in healthy controls (18). These observational studies suggest that gut dysbiosis may be a risk factor for breast cancer development.

The gut microbiome significantly influences the efficacy of multiple anticancer therapies, including chemotherapy, endocrine therapy, and immunotherapy. In HER2-positive breast cancer mouse models and patients, gut microbiota influence the antitumor efficacy of trastuzumab. Antibiotic treatment or transplantation of antibiotic-treated fecal microbiota impairs trastuzumab activity, through mechanisms involving impaired immune cell recruitment and reduced dendritic cell activation and cytokine release (19). Furthermore, plant bioactive compounds such as flaxseed lignans can enhance the anticancer effects of PD-1/PD-L1 inhibitors against breast cancer by modulating the gut microbiota and host immunity (20).

To establish a causal relationship between gut microbiota and breast cancer, Mendelian randomization analysis provides new research evidence. The study reveals causal relationships between gut microbial communities and breast cancer prognosis and risk. For instance, increased abundance of Genus *Sellimonas* is causally associated with elevated risk of ER+ breast cancer, while increased abundance of Genus *Adlercreutzia* exerts a protective effect against ER+ breast cancer (21, 22). By employing genetic variation as instrumental variables, Mendelian randomization studies mitigate confounding factors and reverse causality issues common in traditional observational research, providing stronger evidence for causal pathways within the gut-breast axis.

Although existing research has revealed multifaceted connections between gut microbiota and breast cancer, studies on the differential roles of the gut-breast axis across breast cancer subtypes and its potential impact in disease early stages remain limited. Furthermore, significant gaps persist in understanding how gut microbiota modulate breast cancer development through specific pathways and how to achieve precise microbial interventions. These areas warrant urgent further exploration.

### 3.2 Mechanistic studies

The specific mechanisms by which the gut-breast axis influences breast cancer primarily involve three aspects: estrogen metabolism regulation, immune system modulation, and metabolite-mediated effects.

Elevated levels of endogenous estrogen in the blood increase the risk of breast cancer in women (23). The gut microbiota, particularly the “estrogen biota,” encodes enzymes such as β-glucuronidase. These enzymes can deconjugate conjugated estrogens that have been bound and prepared for excretion by the host liver. This process reactivates the estrogens, allowing them to be reabsorbed by the gut and thereby increasing circulating levels of active estrogens in the body and affecting the responsiveness of breast cancer to endocrine therapy (24–29). The gut microbiota also engages in complex interactions with environmental endocrine disruptors and phytoestrogens. Collectively, these factors influence sex hormone balance, thereby affecting the occurrence, progression, and treatment of hormone-dependent breast cancer (24).

As a key regulator of host immune system development and function, gut dysbiosis can trigger systemic inflammatory responses, affecting immune cell infiltration and function within the tumor microenvironment, thereby promoting or suppressing breast cancer progression (25, 30–33). For example, TLR5-dependent commensal bacteria drive mobilization of myeloid-derived suppressor cells by increasing systemic IL-6 levels, thereby promoting distant malignant tumor progression (34). Chemotherapy-induced gut microbiota alterations correlate with systemic inflammation and weight gain, suggesting gut microbiota may influence chemotherapy side effects by inducing systemic inflammation (35).

The gut microbiota enters the host circulatory system by producing various bioactive metabolites, playing a key role in the gut-breast axis and influencing the physiological state of breast cells and tumor development. Butyrate, produced by gut bacteria fermenting dietary fiber, offers benefits such as maintaining intestinal barrier integrity, regulating immune function, and suppressing inflammation. In breast cancer, it acts as a histone deacetylase inhibitor to reduce LRP5 expression and activate ZFP36, accelerating LRP5 mRNA degradation to block the Wnt/β-catenin signaling pathway, thereby suppressing breast cancer stemness and exhibiting anticancer potential (36). Bile acids are further metabolized by gut microbiota into secondary bile acids, which activate intestinal or systemic receptors (e.g., FXR, TGR5) to influence cell proliferation, apoptosis, and inflammatory responses, thereby affecting breast cancer development (26). Additionally, other metabolites such as tryptophan metabolites and compounds with estrogen-like effects may also influence breast cancer risk (37). Although studies have revealed the potential roles of these metabolites in breast cancer, their specific mechanisms and interactions with other physiological processes require further exploration to develop novel prevention and treatment strategies.

Notably, the gut microbiota also serves as a key interface for the gut-brain axis, translating psychological stress into biological changes that may influence breast cancer. The axis operates via two primary, interconnected mechanisms: systemic immunosuppression and enhanced tumor cell aggressiveness. Immunosuppression is driven in part by the stress-associated loss of *Blautia*, which curtails the production of acetate. This short-chain fatty acid is a crucial metabolic substrate for CD8^+^ T cells, and its deficiency impairs their acetyl-CoA metabolism, compromising critical effector functions like IFN-γ production and ultimately blunting anti-tumor cytotoxicity (38). Concurrently, stress-mediated inhibition of *Bifidobacterium* disrupts the microbial catabolism of oleic acid, leading to its pathological accumulation in the host system, which in turn augments the metastatic capacity of breast cancer cells (39). Thus, the gut-brain axis integrates psychological stress into breast cancer pathophysiology via concrete and targetable host-microbiota metabolic circuits.

### 3.3 Microbiota intervention therapy research

Based on understanding of the gut-breast axis mechanism, various microbiota intervention strategies are being explored for breast cancer prevention and treatment.

Research indicates that probiotics and other therapies aimed at controlling the gut microbiome through bacterial means may aid in breast cancer prevention and even treatment (25). Supplementation with specific probiotics such as *Akkermansia muciniphila* or butyrate can reverse psychostress-induced breast cancer stemness (36); *Lactobacillus plantarum* reduces mammary tumors in Vitamin D receptor (VDR) -deficient mice, suggesting probiotics may exert antitumor effects by regulating intestinal barrier function and inflammatory responses (40). Regarding clinical treatment, the gut microbiome influences the therapeutic efficacy of trastuzumab in HER2-positive breast cancer. Non-responders exhibit lower gut microbial diversity. Transplanting fecal microbiota from responders into mice reproduces the response to trastuzumab, suggesting probiotics may enhance anticancer treatment outcomes by modulating immune responses (19).

Antibiotics can broadly or specifically eliminate microorganisms, thereby altering microbial community composition. Antibiotics intervention studies in breast cancer have found that combining antibiotics with traditional anticancer therapies may enhance treatment efficacy by modulating the microbiome. For example, co-administration of ampicillin with paclitaxel improves chemotherapy effectiveness (41). However, broad-spectrum antibiotic use may cause gut dysbiosis and generate drug resistance, necessitating the development of more specific microbe-targeting strategies.

Diet, as a primary environmental factor shaping gut microbiota composition and function, can modulate the gut microbiome through dietary pattern adjustments or supplementation with specific dietary components, thereby influencing breast cancer progression (33, 42, 43). The pro-tumor effects induced by high-fat diets can be mimicked by transplanting high-fat diet-derived microbiota into control-diet mice, suggesting that diet modulates breast microbiota and tumorigenesis via gut-to-breast signaling pathways (44). Conversely, flaxseed lignans are converted by gut microbes into enterolactones, which suppress breast cancer malignancy by downregulating CD38 and enhance the anticancer effects of PD-1/PD-L1 inhibitors. This mechanism involves regulating the gut microbiota (e.g., increasing *Akkermansia* abundance) and host immunity (20). These studies suggest that personalized dietary interventions combined with gut microbiota modulation hold promise as effective strategies for breast cancer prevention and adjuvant therapy.

## 4 Microbial influence on breast cancer within the tumor microenvironment

### 4.1 Composition of intratumoral microbiota

The advent and refinement of microbial profiling technologies—from traditional culture to high-throughput sequencing and spatial omics—have been pivotal in challenging the dogma of sterile breast tissue. These advances have enabled the precise characterization of the microbial communities residing within the breast tumor microenvironment, revealing compositions that are distinct from adjacent normal tissue and are closely associated with tumor subtypes.

Breast tumor microbiota is ubiquitous. Seminal work by Nejman et al, 2020 (7), analyzing over 1,500 tumors across cancer types identified breast cancer as harboring a particularly rich and diverse microbiota, predominantly composed of intracellular bacteria that can interact with both cancer and immune cells (7). This establishes that intratumoral microorganisms are not passive bystanders but active participants in the tumor ecosystem. For instance, during metastatic colonization, these resident bacteria can enhance host cell survival by remodeling the cytoskeleton to withstand fluid shear stress (45). The routes of bacterial colonization in tumors, potentially involving chemoattraction to necrotic areas, “leaky” vasculature, or local immune suppression, remain an active area of investigation, as do the precise origins and functional roles of intracellular bacteria in breast oncogenesis (46, 47).

Significant differences in microbial abundance and diversity exist between breast tumor tissues and non-cancerous tissues (including normal breast tissues, benign breast disease tissues, and peritumoral tissues), and these differences also vary among different breast cancer phenotypes (48, 49). Most existing studies suggest that *Proteobacteria* is the predominant phylum in breast cancer tissues, while *Actinobacteria*, *Firmicutes*, and *Bacteroidetes* are also detected. Notably, *Actinobacteria* has a higher relative abundance in normal breast tissues (7, 49, 50). Breast cancer tissues have higher abundances of *Enterobacteriaceae*, *Staphylococcus*, and *Bacillus*. In female breast cancer tissues, tumor grade is positively correlated with the relative abundance of *Streptococcus (*7, 49, 51). It is worth noting that compared to patients with benign breast diseases (such as breast nodules) without cancer cells, breast cancer patients have reduced *Proteobacteria* and increased *Firmicutes* in normal breast fat tissues (51). Regarding microbial diversity, current research findings are not consistent. In studies comparing tumor breast tissues with healthy adjacent paired tissues from the same female patients, healthy tissues have higher amplicon sequence variant (ASV) counts than tumor tissues (14). However, other studies have reached the opposite conclusion, with higher bacterial loads and richness in breast tumor samples than in normal breast samples from healthy subjects. Peritumoral normal breast tissues have intermediate bacterial loads and richness between breast tumors and normal samples (7). Specific microbial populations also differ among different molecular subtypes of breast cancer. *Sphingomonas* is enriched in triple-negative breast cancer (TNBC), while *Thermus* dominates in HER2-positive breast cancer (52). TNBC patients who respond to neoadjuvant chemo-immunotherapy (Chemo-IM) have higher intratumoral microbial diversity and burden, suggesting that intratumoral microbiota may be associated with treatment response (17). To date, systematic investigations into microbial biomarkers across different breast cancer subtypes are still scarce, and we anticipate more work will be devoted to this field in the future.

### 4.2 Oncopromotion: facilitating tumor invasion and immunosuppression

Microbiota within the tumor microenvironment promote the occurrence, development, and metastasis of breast cancer through multiple mechanisms, including facilitating tumor invasion and metastasis, inducing immunosuppression, and influencing tumor cell metabolic reprogramming.

Microbes and their metabolites promote tumor growth and metastasis by acting on cancer cells. *Fusobacterium nucleatum* binds to tumor-associated glycosylated antigens to achieve specific colonization in mouse breast tumors through the interaction of its surface Fap2 lectin with Gal-GalNAc on the tumor cell surface (53). Notably, in gastrointestinal tumors, *Fusobacterium nucleatum* and its metabolites bind to tumor cell surface glycosylated antigens, thereby promoting tumor growth and metastasis through pathways such as inducing PD-L1 expression and secreting IL-8 and CXCL1. This suggests that we should pay more attention to the important role of glycosylation in the impact of microbiota on breast cancer in future breast cancer research (54, 55). Enterotoxigenic *Bacteroides fragilis* (ETBF) secretes *Bacteroides fragilis* toxin (BFT), which induces breast epithelial hyperplasia and promotes tumor cell growth and metastasis, possibly by activating the β-catenin and Notch1 pathways (56). Bacterial quorum-sensing peptides can also promote breast cancer cell invasion and angiogenesis (57). Moreover, during the metastatic colonization of breast cancer cells in the lung, intratumoral bacteria carried by circulating tumor cells reorganize the host cell cytoskeleton to enhance resistance to fluid shear stress, thereby promoting host cell survival (45).

The microbiota is a key regulator of the tumor immune microenvironment. Prior work from our group has demonstrated that host cells within the tumor microenvironment can release various factors to mediate tumor immune evasion, while a similar functional capacity has been observed in intratumoral microbiota (58–61). Studies have shown that intratumoral *Haemophilus* promotes tumor progression in 4T1 tumor-bearing mice, weakens the therapeutic effect of αPD-1 monoclonal antibody, and is associated with increased Treg cell infiltration and decreased CD8+ T cell infiltration (62). *Fusobacterium nucleatum* has been found to activate human immune checkpoint receptors TIGIT and CEACAM1 to suppress antitumor immunity in other cancer types, and a similar phenomenon of decreased tumor-infiltrating T cells has been observed in breast cancer after inoculation with *Fusobacterium nucleatum* (53, 63, 64). TLR5-dependent commensal bacteria increase systemic IL-6 levels, driving MDSC mobilization and inhibiting antitumor immunity (34).

Microbiota also influence breast cancer progression through metabolic reprogramming. Nejman et al. found that breast cancer subtypes characterized by increased oxidative stress are enriched with bacteria that produce mycolic acid thiol esters, suggesting that microbiota may adapt to the tumor microenvironment by affecting redox balance (7). There is a close relationship between intratumoral microbiota characteristics and host metabolic heterogeneity. For example, the abundance of specific bacterial phyla (e.g., *Acidobacteria*, *Firmicutes*) correlates with the levels of metabolites in the host microenvironment, such as betaine, with lipids showing a particularly strong association. This suggests that the intratumoral microbiota may influence tumor progression by modulating the metabolic state of host cells or interacting with host metabolites (65). Breast cancer cells have highly plastic metabolic states, and certain microbial genera are significantly associated with cancer metabolic activities. For instance, in breast tumors with low bile acid metabolism—a metabolic state linked to poor prognosis—specific genera such as *Lactobacillus*, *Ruegeria*, and *Marichromatium* are enriched and their presence is strongly associated with the upregulation of nearly all hallmark cell proliferation-related gene sets (66). Furthermore, mechanistic studies reveal a direct causal link: intratumoral colonization by *Sphingobacterium multivorum* leads to a significant decrease in the level of propionylcarnitine, a host-derived metabolite, within the tumor. This depletion of propionylcarnitine contributes to an immunosuppressive microenvironment and promotes tumor progression (62). Cluster analysis based on microbial abundance and tumor metabolic data shows dysregulation of metabolic pathways among different cell types, indicating that microbiota may influence tumor progression and treatment response by regulating host metabolism (67).

### 4.3 Anticancer: enhancing immune surveillance

Despite many studies revealing the oncogenic effects of microbiota in the tumor microenvironment, evidence also suggests that certain microbes or their metabolites have anticancer potential by modulating the host immune system and enhancing antitumor immune responses.

Study revealed that *Anaerococcus*, *Caulobacter*, and *Streptococcus*, which serve as key bacterial hubs in the microbiome–immune network of benign breast tissue, were absent in their cancer-associated counterparts. Moreover, the depletion of *Propionibacterium* and *Staphylococcus* in tumors is negatively correlated with oncogenic immune signatures, while *Streptococcus* and *Propionibacterium* are positively correlated with T cell activation-related genes, indicating that these beneficial bacteria may inhibit tumor development by maintaining local immune homeostasis (68).

The presence of *Stenotrophomonas maltophilia* (SMA) is positively correlated with tumor-infiltrating immune cells, particularly CD8+ T cells. *In vivo* experiments have confirmed that SMA can inhibit tumor growth and promote CD8+ T cell infiltration, suggesting that SMA may exert anticancer effects by enhancing tumor immunity (69). These studies reveal the potential of specific microbes in regulating the functions of immune cells in the tumor microenvironment and provide a basis for developing immune-enhancing strategies based on microbiota.

### 4.4 Clinical translation of microbiome research: from diagnostic biomarkers to targeted therapies

#### 4.4.1 The intratumoral microbiome as a novel class of predictive biomarkers

Oncology research has always been committed to finding more accurate and stable markers for cancer early screening and prognostic prediction assessment, such as imaging, pathology, circulating tumor cells, and serum biomarkers (61). Intratumoral microbiota can serve as a powerful and specific predictor of the response of TNBC patients to neoadjuvant chemo-immunotherapy. The pathological complete response group has higher intratumoral microbial diversity and burden, which is associated with increased T cell infiltration and reduced tumor-associated macrophage infiltration, indicating that intratumoral microbiota is closely related to the immune cell composition of the tumor microenvironment and can influence patients’ responses to combined therapies (17). By integrating microbiome data with clinical information, AI diagnostic models are expected to enable early diagnosis, prognostic prediction, and assessment of treatment responses for breast cancer, thereby promoting the development of personalized medicine. However, the development of AI models requires large-scale, multicenter, and prospective studies to verify their stability and generalizability and to further clarify the causal relationship between the microbiome and breast cancer pathophysiology.

#### 4.4.2 Emerging microbiome-targeted therapeutic interventions

Based on a deep understanding of the interactions between the microbiota in the tumor microenvironment and breast cancer, researchers are actively exploring the application of microbiomics in clinical treatment strategies for breast cancer, including targeted microbial clearance, strain engineering, and combination with immunotherapy.

##### 4.4.2.1 Targeted depletion of oncogenic microbes

This strategy focuses on the selective elimination of tumor-promoting bacteria to inhibit tumor growth and metastasis. *Fusobacterium nucleatum* is commonly found in breast cancer and creates an immunosuppressive tumor microenvironment characterized by high myeloid cell influx, hindering immune checkpoint blockade therapy. Administration of the antibiotic metronidazole in breast cancer can clear *Fusobacterium nucleatum* and reshape the tumor microenvironment. To avoid the destruction of systemic microbial community diversity and abundance by broad-spectrum antibiotics, researchers have designed a biomimetic nanocarrier that can precisely home to breast cancer and effectively eliminate intratumoral *Fusobacterium nucleatum* without disturbing the systemic microbiota, significantly improving the therapeutic effect of PD-L1 blockade and prolonging survival (70). Similarly, nanoplatforms coated with bacterial outer membrane vesicles have been engineered to simultaneously target and eliminate *F. nucleatum* and cancer cells, effectively converting intratumoral bacteria into *in-situ* immune potentiators to reverse the “cold” tumor phenotype in triple-negative breast cancer (TNBC) (71).

##### 4.4.2.2 Engineered microbial therapeutics

Strain engineering involves using synthetic biology techniques to genetically edit microbes to endow them with specific antitumor functions, such as secreting anticancer substances, activating immune responses, or serving as drug delivery vehicles. Microbial genome regulation and engineered microbial enzymes have certain application potentials in breast cancer management (3). Referring to the applications of this field in other cancer therapies and combining with live bacteria tracing technologies, future studies may enable real-time *in vivo* tracing of engineered strains to evaluate their colonization, proliferation, and functional expression within tumors, thereby optimizing treatment plans.

## 5 Summary and future perspectives

This review systematically summarizes the significant role of the human microbiome in the occurrence and progression of breast cancer, highlighting the broad prospects of microbe-host interactions in breast cancer research from technological innovations to mechanistic explorations and clinical translational applications.

Current research has established that breast tissue is not a sterile environment but harbors a unique microbial community. Gut microbiota regulates estrogen metabolism, immune function, and metabolite production via the “gut-breast axis,” thereby influencing breast cancer risk and treatment response. Microbiota within the tumor microenvironment participate in breast cancer progression through mechanisms such as promoting invasion, inducing immunosuppression, and metabolic reprogramming, while certain microbes with anticancer potential also exist.

Future research should focus on the following directions: in-depth elucidation of the causal mechanisms by which microbes colonize and influence breast cancer, using techniques such as gene editing and organoid models to verify the functions of specific strains; development of more precise microbe-targeted intervention strategies, such as engineered strains and nanocarrier delivery systems, to enhance therapeutic specificity and reduce side effects; large-scale prospective clinical studies to validate the clinical value of microbial biomarkers in breast cancer diagnosis, prognostic prediction, and treatment guidance; exploration of the synergistic mechanisms between the microbiome and existing therapeutic modalities (chemotherapy, radiotherapy, immunotherapy) to develop new combination therapies; and strengthening of multidisciplinary collaborations to integrate technologies from microbiomics, immunology, metabolomics, and artificial intelligence, thereby advancing the precise prevention and treatment of breast cancer.

As research continues to advance, the microbiome holds promise as a novel target for breast cancer prevention and treatment, providing significant opportunities for the development of new therapeutic strategies and the realization of personalized precision medicine.
